# Tumor immunology: a short IL18 cleavage product promotes cancer immunosurveillance

**DOI:** 10.1038/s41392-025-02213-y

**Published:** 2025-04-23

**Authors:** Emma Guilbaud, Lorenzo Galluzzi

**Affiliations:** Cancer Signaling and Microenvironment Program, Fox Chase Cancer Center, Philadelphia, PA USA

**Keywords:** Tumour immunology, Inflammation, Innate immune cells

In a recent study published in *Nature Immunology*, Shen et al. have proposed that the caspase 3 (CASP3)-dependent cleavage of interleukin 18 (IL18) in malignant cells exposed to apoptotic inducers generates a short IL18 fragment that operates intracellularly to promote the secretion of IFN-stimulated gene 15 (ISG15) ubiquitin-like modifier, culminating with the recruitment of natural killer (NK) cells to the tumor microenvironment in support of cancer immunosurveillance.^1^ While these findings delineate a novel signaling pathway linking atypical proteolytic processing of IL18 by malignant cells to anticancer immunity, the actual implication of CASP3 in such a signal transduction cascade remains unclear.

Malignant cells form clinically manifest neoplasms once they acquire (epi)genetic alterations that enable them to evade anticancer immunity.^2^ Such an immunoevasive phenotype can emerge from three conceptually distinct, although at times partially overlapping, scenarios (recently dubbed as the “three Cs”): (1) camouflage, referring to the ability of neoplastic cells to avoid being localized or detected by immune effector cells; (2) coercion, describing the capacity of transformed cells to interfere with immune cell functions; and (3) cytoprotection, denoting the increased resistance of cancer cells to cytotoxic immune effectors.^2^

Coercion has been associated with changes in the surface properties of malignant cells, as well as with alterations in the cancer cell secretome.^2^ The latter generally involves an augmented secretion of immunosuppressive factors like C-C motif chemokine ligand 2 (CCL2), coupled with a diminished release of immunostimulatory molecules such as type I interferon (IFN).^2^ Importantly, apoptotic CASP3, which is generally activated by a supramolecular CASP9-containing supramolecular platform commonly referred to as apoptosome, promotes coercion by a number of different mechanisms (including the proteolytic inactivation of pro-inflammatory proteins), most likely reflecting the notion that apoptosis is central for physiological (post-)embryonic development and adult tissue homeostasis, and hence must not elicit inflammation.^3,4^ Conversely, inflammatory caspases including CASP1 generally antagonize coercion, as their activation within multiprotein complexes commonly known as inflammasomes promotes the proteolytic maturation and release of pro-inflammatory cytokines such as interleukin 1 beta (IL1B) and IL18.^3,4^

Shen and collaborators set to study the ability of apoptotic caspases to cleave IL18, demonstrating both in vitro and *in cellula* (harnessing human HEK293T cells exposed to apoptotic conditions) that CASP3 (but not CASP7, CASP8 or CASP9) generates a small (~15 kDa) IL18 fragment upon cleavage after D69 (in mouse systems) or D71/D76 (in human systems), as demonstrated by D→A mutagenesis experiments at these sites. Contrary to mature IL18 (mIL18) as generated by the CASP1-dependent processing of full-length IL18 (flIL18), short IL18 (sIL18) as produced by the CASP3-dependent cleavage thereof failed to elicit interferon-gamma (IFNG) secretion by NK cells. Moreover, sIL18 was not found in the supernatant of HEK293T cells or J774 macrophages treated with apoptotic inducers, whereas mIL18 was normally secreted by J774 macrophages in response to inflammasome activators. Subcellularly, sIL18 predominantly localized to the nucleus, despite lacking a bona fide nuclear localization signal, potentially owing to an interaction with nucleoporins.^1^

Next, Shen et al. set to investigate the role of IL18 in cancer immunosurveillance, harnessing mouse HR^+^ mammary carcinoma EO771 cells and mouse colorectal cancer (CRC) MC38 cells, both of which express flIL18. In these systems, deleting or downregulating *Il18* accelerated tumor growth in vivo, in immunocompetent syngeneic C57BL/6 mice, a detrimental phenotype that could be reversed by the transgene-driven overexpression of wild-type (WT) IL18, as well as by CASP1-resistant IL18^D35A^, but not CASP3-resistant IL18^D69A^ in malignant cells. Similarly, the adenoviral delivery of an IL18-coding gene to mouse melanoma B16-F10 cells (which per se do not express flIL18) limited their growth upon inoculation into WT C57BL/6 mice. Importantly, the ability of WT IL18 to suppress the growth of B16-F10 melanomas in vivo was retained in *Il18r1*^*−/−*^ mice (which lack the main IL18 receptor), further demonstrating that this does not involve pro-inflammatory signaling by mIL18.^1^

B16-F10 cells overexpressing WT IL18 or IL18^D69A^ did not differ from each other in terms of proliferation rate or sensitivity to apoptotic stimuli. Moreover, the ability of WT IL18 to limit the in vivo progression of B16-F10 melanomas was lost in immunodeficient mice. In line with an active involvement from the host immune system, *Il18*^*−/−*^ EO771 and MC38 lesions established in WT C57BL/6 mice exhibited reduced tumor infiltration by NK cells as compared to their control counterparts, which could be partially restored by the transgene-driven overexpression of WT IL18 but not IL18^D69A^. Comparable findings were obtained with B16-F10 cells optionally engineered to express WT IL18 or IL18^D35A^, but not IL18^D69A^, or optionally receiving an IL18-coding adenovirus in vivo. Importantly, the ability of WT and CASP1-resistant IL18^D35A^ to promote NK cell infiltration was retained in *Il18r1*^*−/−*^ mice. Confirming a mechanistic role for NK cells in the antitumor effects of sIL18, IL18-overexpressing B16-F10 melanomas progressed as rapidly as their IL18^D69A^-overexpressing counterparts in C57BL/6 mice subjected to the antibody-dependent depletion of NK cells.^1^

RNA sequencing studies demonstrated that IL18-overexpressing B16-F10 melanomas are enriched in transcripts associated with NK cell activity as compared to their IL18^D69A^-overexpressing counterparts, notably transcripts coding for interferon-stimulated genes (ISGs), an effect that was linked to increased activating phosphorylation of signal transducer and activator of transcription 1 (STAT1). Similar results were obtained in vitro, upon treatment of IL18-overexpressing vs IL18^D69A^-overexpressing B16-F10 cells with tumor necrosis factor (TNF) plus interferon-gamma (IFNG), which promoted rapid and CASP3-dependent cell death, as well as by transducing B16-F10 cells with an sIL18-coding construct. Genetic experiments demonstrated that STAT1 expression is required for the transcriptional programs elicited by sIL18. Alongside, a mechanistic involvement of CASP3 was suggested based on the effects of the pharmacological caspase inhibitor z-DEVD,^1^ which however at the concentration employed (20 μM) inhibits not only a number of caspases beyond CASP3, but also other proteases.

Mass spectrometry studies proved that sIL18 signaling results in the secretion of multiple bioactive factors by B16-F10 cells, including the NK cell activator ISG15. Consistently, sIL18 favored the binding of STAT1 to the *Isg15* promoter, *de facto* eliciting *Isg15* transactivation. Moreover, replenishing ISG15 in the supernatant of B16-F10 cells subjected to the downregulation of STAT1 restored its NK cell-activating properties. Finally, the ability of sIL18 to limit the progression of B16-F10 melanomas established in WT C57BL/6 mice was abolished with the administration of an antibody blocking the ISG15 receptor integrin alpha L (ITGAL, best known as LFA-1α), as well as by the deletion of *Isg5* in malignant cells.^1^

Shen and co-authors went on to focus on the molecular mechanisms underlying STAT1 activation by sIL18, which (1) per se lacks kinase activity, (2) was found not to directly bind STAT1, (3) failed to alter STAT1 nuclear translocation. Immunoblotting studies harnessing pharmacological inhibitors pointed to cyclin-dependent kinase 8 (CDK8), which had previously been shown to promote STAT1 activation,^5^ as a potential signal transducer in the cascade of events connecting IL18 cleavage to ISG15 secretion. Accordingly, silencing *Cdk8* limited STAT1 activation and *Isg15* upregulation as elicited by sIL18 in B16-F10 cells. Mechanistically, sIL18 was shown to bind the catalytic domain of CDK8, resulting in heightened kinase activity but no alterations in CDK8 binding to STAT1.

**Fig. 1:**
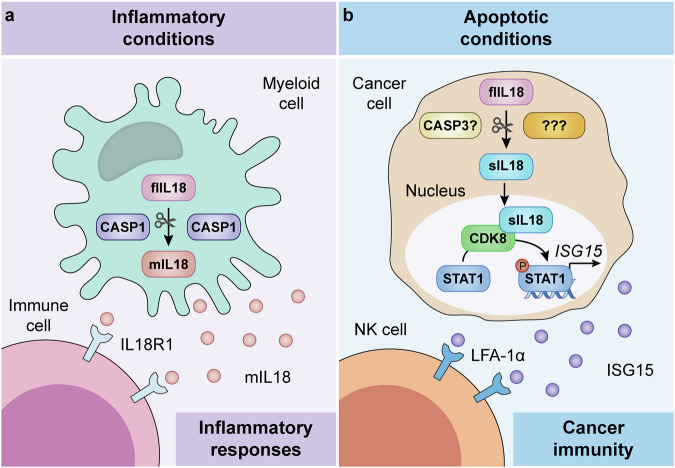
A novel signaling pathway linking IL18 proteolytic processing to anticancer immunity. In some cells including monocytes and macrophages, the proteolytic processing of immature interleukin 18 (IL18) by caspase 1 (CASP1) results in the generation of mature IL18 (mIL18) and its secretion, resulting in the establishment of inflammatory conditions upon binding to interleukin 18 receptor 1 (IL18R1) on target cells (**a**). Recent data demonstrate that—in some settings—malignant cells expressing full-length IL18 (flIL18) can cleave it at an alternative site (which is also a substrate for CASP3, at least in vitro), resulting in the formation of an atypical IL18 fragment that is not secreted. Short IL18 (sIL18) instead appears to bind cyclin-dependent kinase (CDK8) to promote signal transducer and activator of transcription 1 (STAT1) functions, culminating with the release of numerous bioactive factors including ISG15 ubiquitin-like modifier (ISG15) and the consequent recruitment of natural killer (NK) cells to the tumor microenvironment in support of cancer immunosurveillance (**b**). The actual mechanistic contribution of CASP3 (*vs* other proteases activated in apoptotic conditions that may recognize the same cleavage site) to the generation of sIL18 in malignant cells forming progressive neoplasms in vivo, however, remains to be formally established. LFA-1α (official name: ITGAL) integrin alpha L, P inorganic phosphate

In summary, Shen et al. documented a novel signal transduction cascade linking the generation of an atypical IL18 fragment to the CDK8-dependent activation of STAT1 and the consequent establishment of a transcriptional program dominated by ISG15 that sustains tumor-targeting immune responses (Fig. 1). Suggesting a potential implication of these findings in humans, patients with CRC exhibiting higher-than-median nuclear IL18 (which in CRC cell lines appeared to be exclusively accounted for by sIL18), as assessed in malignant cells by immunohistochemistry, were reported to experience superior overall survival, especially if high nuclear sIL18 was accompanied by elevated STAT1 phosphorylation.^1^

That said, CASP3 activation has been mechanistically associated with immunoevasion through a number of distinct mechanisms in various preclinical tumor models (including MC38 CRCs), and whether CASP3 is indeed responsible for sIL18 generation in vivo remains to be formally established. Indeed, in vivo experiments by Shen et al. largely relied on the use of IL18^D69A^, which may resist cleavage by proteases other than CASP3 that are also activated by apoptotic stimuli. In vivo experiments with *Casp3*^*−/−*^ cancer cells are therefore urgently awaited to clarify the actual implication of CASP3 in these findings.
